# Metabolic Reprogramming of the Liver by IL-33: A Protective Response to Acute Injury

**DOI:** 10.3390/cells15151346

**Published:** 2026-07-27

**Authors:** Ying Wu, Weinan Gao, Mengrui He, Wenda Zhang

**Affiliations:** Department of Pathophysiology, College of Basic Medical Sciences, Jilin University, Changchun 130021, China; wuying22@mails.jlu.edu.cn (Y.W.); gaown23@mails.jlu.edu.cn (W.G.); hemr23@mails.jlu.edu.cn (M.H.)

**Keywords:** IL-33, glucose metabolism, acute liver injury

## Abstract

**Highlights:**

**What are the main findings?**

- IL-33 enhances mitochondrial oxidative phosphorylation metabolism in the liver via PDK4.
- Metformin increases the expression of IL-33 in liver tissue and alleviates acute liver injury induced by CCl_4_.

**What is the implication of the main finding?**

- This study uses the metabolic characteristics of liver cells with overexpressed IL-33 to more deeply elucidate the mechanism by which metformin affects liver cell metabolism, providing a new theoretical basis for the clinical use of metformin to suppress inflammation and protect the liver, and serving as a new molecular target for the treatment of liver diseases.

**Abstract:**

Background and Aims: The glucose metabolism pattern of the liver is closely related to liver diseases. In recent years, alcoholic hepatitis, viral hepatitis, fatty liver and other diseases have become important liver diseases that impact people’s lives. IL-33 (interleukin-33), as a member of the IL-1 cytokine family, and “alarmin” play a multi-dimensional regulatory role in various physiological and pathological processes of the liver. Methods: This study, by integrating transcriptomic data analysis and in vitro and in vivo experiments, expounds on new insights into the regulation of hepatocyte fate by IL-33 through metabolic reprogramming and reveals the potential, new role of IL-33 as a regulatory factor of metabolism. Conclusions: Our research further confirmed that IL-33, in addition to serving as an alarm hormone, is also a key checkpoint for liver metabolism, capable of promoting oxidative phosphorylation in hepatocytes and antagonizing acute liver injury induced by CCl_4_.

## 1. Introduction

The liver, as a metabolic hub, regulates the metabolism of carbohydrates, lipids and amino acids [1,2]. Liver metabolic reprogramming denotes the adaptive alterations by which hepatocytes and nonparenchymal cells (for example, hepatic stellate cells and immune cells) systematically remodel core metabolic pathways—glucose, lipid, and amino acid metabolism—to accommodate changes in internal and external conditions (such as injury, inflammation, nutritional stress, and the tumor microenvironment) [3,4,5]. Liver metabolic reprogramming and mitochondrial function are tightly and bidirectionally regulated. Mitochondria serve not only as central hubs of energy metabolism but also as critical integrators of metabolic signals that influence cell fate [6]. Mitochondrial dysfunction drives liver metabolic reprogramming [7,8]. The influence of liver metabolic reprogramming on injury outcomes depends on the type and severity of external stressors. In particular, the interplay between systemic responses and local hepatic metabolism during injury remains poorly understood. Thus, integrating both internal and external models is necessary to elucidate how metabolic reprogramming drives liver injury.

IL-33, a member of the IL-1 cytokine family, has recently been recognized not only as an “alarmin” [9] but also as an important regulator of cellular metabolic reprogramming [10]. Increasing evidence indicates that IL-33 contributes significantly to systemic metabolic disorders, including type 2 diabetes [11] and heart disease [12]. In addition, IL-33 can antagonize the Warburg effect (aerobic glycolysis), promote the oxidative decomposition of pyruvate in retinal pigment epithelial (RPE) cells, and maintain mitochondrial respiratory function [13]. When IL-33 is deficient, mitochondrial function declines, morphology is altered, and cellular bioenergetics shift predominantly toward glycolysis, compromising functional stability. However, in liver disease, there remains controversy about whether IL-33 acts as a proinflammatory or anti-inflammatory mediator across distinct pathological contexts and cell types, and its relationship to hepatic metabolic patterns remains unclear [14,15,16,17,18,19]. Therefore, exploring the role of IL-33 (interleukin-33) as an “alarmin” in the relationship between liver metabolism and liver function protection will fill the gap between liver inflammation and metabolism.

Pyruvate dehydrogenase kinase (PDK), which is highly expressed in the liver [20], inhibits the pyruvate dehydrogenase complex (PDH) through phosphorylation, blocking pyruvate from entering the tricarboxylic acid cycle and promoting the shift of cellular metabolism from oxidative phosphorylation to glycolysis [21]. This metabolic regulatory node plays a key role in a variety of liver diseases [22]. Previous studies have found that in a mouse model of acute liver injury induced by concanavalin A (Con A), the expression of PDK2 is significantly associated with the progression of liver injury [23]. Excessive activation of PDK is a key metabolic link in alcohol-related liver diseases, and DCA exerts a significant liver-protective effect by inhibiting PDK [24]. PDK is an important target that links metabolic reprogramming and inflammatory responses in liver diseases (especially acute injury and alcoholic liver disease). Pyruvate dehydrogenase kinase regulates macrophage polarization in metabolic and inflammatory diseases [25]. However, whether liver metabolism regulated by IL-33 is related to PDK has not been studied.

## 2. Material and Methods

### 2.1. Analyze the Interacting Proteins of IL-33 and PDK4 Using the String Online Database

The String online database (https://cn.string-db.org/, accessed on 14 April 2026) was used to analyze the interacting proteins of IL-33 and PDK4 and their gene enrichment processes.

### 2.2. Transcriptomic Data Analysis

Transcriptomic profiling was performed by Paisenuo Biotechnology Co., Ltd. (Shanghai, China). We processed the returned data by first trimming raw reads with Cutadapt to obtain high-quality sequences, which were then aligned to the mouse reference genome. Gene-level expression was quantified from the alignment results. We then conducted differential expression and enrichment analyses on mouse liver tissue. Data visualizations were generated using the Microbioinformatics online platform.

(1) The DESeq software (version 1.44.0) was used to conduct differential analysis of gene expression. The criteria for screening differentially expressed genes were as follows: a fold change in expression |log2FoldChange| > 1 and a significant *p*-value < 0.05. The gplots2 package in R language (version 4.3.1)was used to draw the volcano plot of differentially expressed genes.
(2) The Blast2go software (version 5.2.5) was used for GO enrichment analysis (database: Gene Ontology, http://geneontology.org/). During the analysis, the gene list and the number of genes for each GO term were calculated using the differentially expressed genes annotated by GO terms. Then, the *p*-value was calculated by the hypergeometric distribution method (the criterion for significant enrichment was *p*-value < 0.05). The GO terms in which the differentially expressed genes were significantly enriched compared with the entire genomic background were identified to determine the main biological functions of the differentially expressed genes.
(3) KEGG enrichment analysis was performed using clusterprofiler (database: Kyoto Encyclopedia of Genes and Genomes, http://www.kegg.jp/, version 4.8.3). During the analysis, the gene list and the number of genes in each pathway were calculated using the differentially expressed genes annotated by KEGG pathway. Then, the *p*-value was calculated by the hypergeometric distribution method (the criterion for significant enrichment was *p*-value < 0.05). KEGG pathways in which the differentially expressed genes were significantly enriched compared with the whole-genome background were identified, so as to determine the main biological functions of the differentially expressed genes.

### 2.3. Animal Sources

SPF-grade wild-type C57BL/6 mice were purchased from Beijing Vital River Laboratory Animal Technology Co., Ltd. IL33 transgenic mice were kindly provided by Prof. Ying Sun from Capital Medical University (Beijing, China). The construction of IL33 transgenic mice is detailed in reference [26]. The mice were housed in the Experimental Animal Center of the Basic Medical College of Jilin University. The animal experiments in this paper were reviewed by the Ethical Review Committee of the Basic Medical College of Jilin University (NO.2021501). The experiments followed the 3R principles for the use of experimental animals and provided humane care, complying with relevant national regulations on the welfare of experimental animals.

### 2.4. Animal Grouping

Forty 6–8-week-old wild-type C57BL/6 mice and forty 6–8-week-old C57BL/6 mice with IL-33 gene overexpression were randomly divided into five groups, with 8 mice in each group. The groups were as follows: the normal saline control group (Vehicle 1 group, 10 mL/kg), the olive oil group (Vehicle 2 group, 10 mL/kg), the metformin group (Met group, 200 mg/kg, dissolved in normal saline), the carbon tetrachloride model group (CCl_4_ group, 0.3%, 10 mL/kg, dissolved in olive oil), and the metformin pre-treatment group (Met + CCl_4_ group, Met 200 mg/kg, CCl_4_ 0.3% 10 mL/kg). In the metformin pre-treatment group, metformin was administered 30 min before the administration of CCl_4_. All drugs were administered via intraperitoneal injection.

### 2.5. Cell Source

AML12 (Haixing Biology, Suzhou, China) was cultured in DMEM supplemented with 10% FBS, 1% P/S, 0.5% ITS—G, and 40 ng/mL dexamethasone at 37 °C in an atmosphere containing 5% CO_2_.

### 2.6. Cell Transfection

A total of 2.0 μg of pcDNA3 was used. 1-CMV-MCS-SV40-Purowas IL-33 was transfected in OptiMem medium (Gibco, Waltham, MA, USA) using TurboFect Transfection Reagent (Thermo Scientific, Waltham, MA, USA). At 24 h post-transfection, the cells were harvested and assayed for RNA levels of the target of interest.

Forward primer (TTAAGCTTGGTACCGAGCTCGGATCCgccaccatgagacctagaat).

Reverse primer (CAGCGGGTTTAAACGGGCCCTCTAGAcTACTTGTCATCGTCATCCT).

### 2.7. Detection of Cellular Glucose, Lactate, and NAD^+^ Content

Twenty-four hours after the transfection of AML12 cells, the glucose consumption, lactate production, and NAD^+^ content of the cells were detected using a glucose detection kit (Nanjing Jiancheng Bioengineering Institute, Nanjing, China), a lactate detection kit (Nanjing Jiancheng Bioengineering Institute, Nanjing, China), and an NAD^+^ content detection kit (Beyotime Biotechnology, Shanghai, China).

### 2.8. Analysis of the Effects of Metformin on the Liver Using the NCBI Online Database

The dataset GSE90755 regarding the effect of metformin on the liver was screened from the NCBI database. The DESeq software was used to conduct differential analysis of gene expression. The criteria for screening differentially expressed genes were as follows: an absolute value of log2 fold change (|log2FoldChange|) > 1 and a significant *p*-value < 0.05. The online website of Microbioinformatics was used for data graph drawing.

### 2.9. Detection of Serum Aspartate Aminotransferase (AST)/Alanine Aminotransferase (ALT) Activities

Using alanine aminotransferase (ALT) and aspartate aminotransferase (AST) kits (Nanjing Jiancheng Bioengineering Institute, Nanjing, China), according to the kit instructions, mouse serum was collected. The absorbance at 510 nm of each well was measured using a microplate reader. The absolute OD value (OD value of the test well—OD value of the blank control well) was used to query the standard curve, and the corresponding activity unit values of AST/ALT were obtained.

### 2.10. Hematoxylin–Eosin Staining (HE Staining) of Tissue Sections

Mouse liver tissues were fixed with 4% paraformaldehyde and then dehydrated. Paraffin blocks of liver tissues were prepared and sectioned. After drying at 45 °C, the sections were dewaxed with xylene. They were then successively treated with ethanol solutions of different concentration gradients. The sections were stained with hematoxylin solution for 5 min and washed for 2 min. They were treated with 1% hydrochloric acid alcohol for 6 s and washed for 1 min, followed by treatment with dilute ammonia solution for 30 s and washing for 1 min. Eosin staining was performed for 3 min and the sections were washed for 2 min. The sections were dehydrated with ethanol solutions of different concentration gradients and cleared with xylene. Finally, the sections were mounted with neutral resin, observed under a microscope and photographed.

### 2.11. Western Blot

After grinding the frozen tissue in liquid nitrogen, weigh 20 mg and add 200 μL of the prepared RIPA lysis buffer (1% PMSF + 1% β-mercaptoethanol, Beyotime Biotechnology Co., Ltd., Shanghai, China) for lysis. After homogenizing with a tissue homogenizer, let it stand on ice for 20 min. Then, perform sonication, centrifuge and collect the supernatant. Determine the protein concentration using the BCA method. According to the required sample preparation volume, pipette the supernatant into a new EP tube, add the loading buffer, and boil in boiling water for 10 min to prepare the protein sample. Use a 10% separating gel for SDS-PAGE. After the electrophoresis is completed, transfer the membrane using the “sandwich” method. Block with 5% non-fat milk powder at room temperature for 1 h. Incubate with primary antibodies F4/80 (CST, Danvers, MA, USA) and CD06 (CST, Danvers, MA, USA) at 4 °C overnight. Wash the membrane with TBST three times. Incubate with mouse/rabbit anti-goat secondary antibody (Proteintech, Wuhan, China) at room temperature. Wash the membrane with TBST three times. Prepare the chromogenic solution to develop the protein bands on the PVDF membrane.

### 2.12. qPCR Experiment

The RNA of mouse liver tissue was extracted using the TRIZOL method. Subsequently, the extracted RNA was reverse-transcribed using a reverse transcription kit (Yeasen Biotechnology, Shanghai, China). The reactants were prepared in a 20 μL system, and the reaction conditions were as follows: 95 °C for 30 s; denaturation at 95 °C for 5 s; annealing at 50–60 °C for 30 s; and extension at 72 °C for 10 s. The denaturation and annealing steps were repeated for 40 cycles. Finally, the 2^−ΔΔCt^ relative quantification method was used, and the Ct value of β-actin was utilized to quantify the target gene.

The primer sequences are as follows:

IL-33: ACTATGAGTCTCCCTGTCCTG, ACGTCACCCCTTTGAAGC;

ATF4: CCTATAAAGGCTTGGGGCCA, TGAAGAGCGCCATGGTTTAG;

ATF5: TCTGGGAACCCCTGTGGATTA, GCAGCGTGGAAGATTGTTCAG;

Ccr1: CTCATGCAGCATAGGAGGCTT, ACATGGCATCACCAAAAATCCA;

Chil3: ACATGGCATCACCAAAAATCCA, ACATGGCATCACCAAAAATCCA;

CAT: GGAGGCGGGAACCCAATAG, GTGTGCCATCTCGTCAGTGAA;

SOD1: ATGGCGATGAAAGCGGTGT, CCTTGTGTATTGTCCCCATACTG;

SOD2: CAGACCTGCCTTACGACTATGG, CTCGGTGGCGTTGAGATTGTT;

PDK2: AAGACGCCTATGACATGGCTA, GCCACCATAATCTTGATGGGG;

PDK4: AGGGAGGTCGAGCTGTTCTC, GGAGTGTTCACTAAGCGGTCA;

IL1RL1: TACCAAATGCCAGTCCCGAA, CTGAGTAGGCAAGTCCACGA.

### 2.13. Immunofluorescence Assay

Prepare liver tissue wax blocks, section them with xylene, and then use a microtome to cut sections and mount them on glass slides. Then, stain the sections with different primary antibodies, F4/80 (CST, Danvers, MA, USA) and CD206 (CST, Danvers, MA, USA), respectively, and then incubate them with the corresponding secondary antibodies according to the manufacturer’s instructions. Observe and analyze the slides using a microscope slide scanner.

### 2.14. Immunohistochemistry Experiment

In this experiment, the streptavidin–biotin–peroxidase complex (SP) method was used for immunohistochemical staining of paraffin tissue sections. The expression localization and level of the target protein IL-33 in the tissues were observed under an optical microscope. The tissues used in the experiment were liver tissue samples after drug treatment. All operations were carried out at room temperature, and the time and temperature of each step were strictly controlled to ensure the stability and reproducibility of the staining results.

### 2.15. Data Statistics

All values are presented as mean ± standard deviation. Statistical analysis was performed using SPSS 22.0 software. One-way analysis of variance was used for comparisons between groups. Analysis and plotting were conducted using GraphPad Prism 8.0 statistical software.

## 3. Results

### 3.1. IL-33 Promotes Mitochondrial Metabolism in the Liver

**Figure 1:**
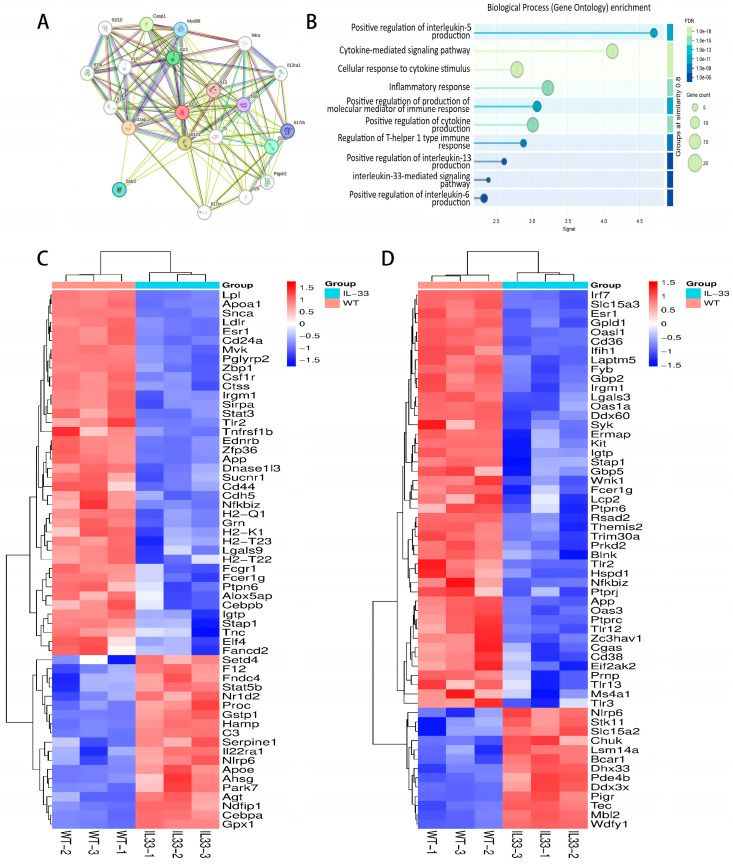
IL-33 affects liver inflammation and immune response. (**A**) Analysis of IL-33 interacting proteins using the STRING database. The interactions among IL-33 and other hub genes by STRING. (**B**) Enrichment pathway analysis of IL-33-interacting proteins (1.0e−18 means 1 × 10^−18^) (**C**) Construction of IL-33 gene overexpression in mice and analysis of differentially expressed genes related to inflammatory response between IL-33-overexpression mice and wild-type mice. (**D**) Differentially expressed genes related to immune response between IL-33-overexpression mice and wild-type mice.

To comprehensively analyze the function of IL-33 in liver metabolic regulation, the String online database (https://cn.string-db.org/, accessed on 14 April 2026) was first used to analyze the interacting proteins of IL-33 (Figure 1A) and their major enriched pathways (Figure 1B). The results showed that the interacting proteins of IL-33 mainly included IL1r1, IL13, IL1rap, Myd88, etc. Further gene enrichment analysis of the interacting proteins revealed that these genes were mainly enriched in cellular responses to cytokine stimuli, regulation of inflammatory responses, and immune pathways.

To further validate the role of IL-33 in the liver, transgenic mice were constructed using IL-33. The results showed that compared with wild-type mice, the differentially expressed genes in IL-33 overexpressing mice were enriched in inflammation (Figure 1C) and immune pathways (Figure 1D). The differentially expressed genes in the inflammation and immune response pathways included IL22ra1, Stat3, Fcgr1, Eif4, CD36, Ptprj, Eif2ak2, etc., suggesting that IL-33 overexpression in mice affects the liver immune inflammatory response through comprehensive multi-pathway intervention.

**Figure 2:**
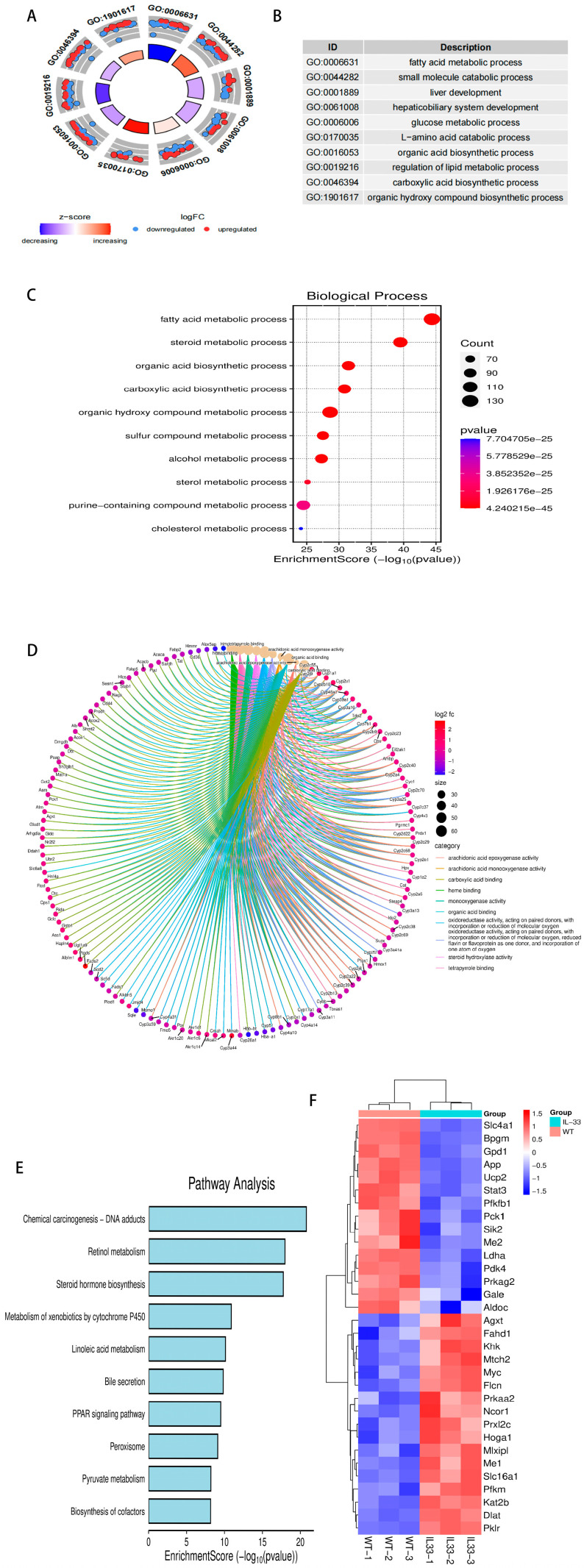
IL-33 affects the liver metabolic process. (**A**) Mice with IL-33 gene overexpression were constructed, and the liver tissues of the mice were extracted to analyze the GO enrichment of differentially expressed genes between IL-33-overexpression mice and wild-type mice. Red indicates upregulation, and blue indicates downregulation. (**B**) Specific GO enrichment processes of differentially expressed genes between IL-33 and wild-type mice. (**C**) Bubble plot of the biological process enrichment of differentially expressed genes between IL-33 and wild-type mice. The size of the bubble represents the number of enriched genes, and the color of the bubble represents the *p*-value (7.704705e−25 means 7.704705 × 10^−25^). (**D**) Molecular function gene-pathway association network diagram of differentially expressed genes between IL-33 and wild-type mice. (**E**) KEGG enrichment of differentially expressed genes between IL-33-overexpression mice and wild-type mice. (**F**) Heat map of the differential expression of genes related to pyruvate metabolism between IL-33-overexpression mice and wild-type mice.

To investigate the role of IL-33 in regulating hepatic immune inflammation, we compared liver transcriptomes from IL-33-overexpressing mice and wild-type controls. Differentially expressed genes predominantly affected pathways of fatty acid, glucose, and amino acid metabolism. Enrichment analysis further highlighted alterations in the pyruvate metabolic process (Figure 2). As a central intermediate of glucose metabolism, pyruvate links glycolysis, the tricarboxylic acid cycle (TCA), gluconeogenesis, lipid synthesis, and amino acid metabolism, and thus plays a pivotal role in hepatic metabolic flux. Gene-level analysis of the pyruvate pathway showed significant downregulation of Ldha and PDK4 and significant upregulation of Dlat and Pklr in IL-33–overexpressing mice, suggesting that IL-33 may modulate hepatic inflammatory responses by reshaping pyruvate metabolism.

### 3.2. IL-33 Promotes Mitochondrial Metabolism in the Liver Through PDK4

**Figure 3:**
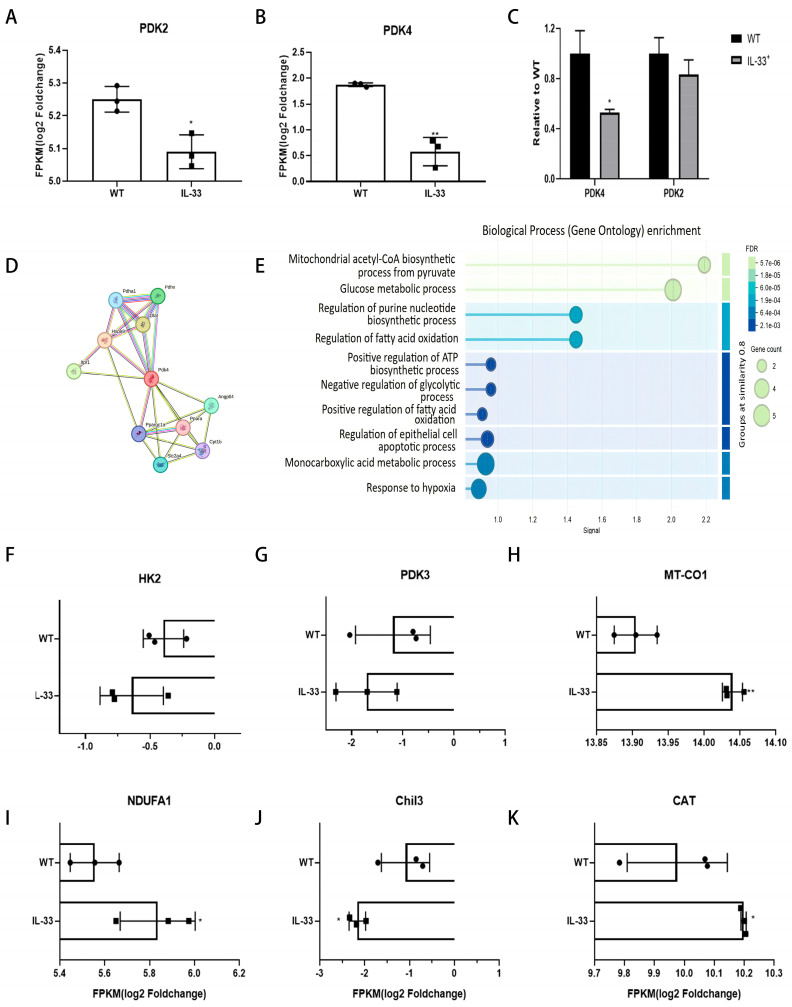
IL-33 promotes mitochondrial oxidative phosphorylation metabolism in the liver. (**A**) Differences in the expression levels of the PDK2 gene in the liver tissue transcriptomics between IL-33-gene-overexpressing mice and wild-type mice. (**B**) Differences in the expression levels of the PDK4 gene in the liver tissue transcriptomics between IL-33-gene-overexpressing mice and wild-type mice. (**C**) Verification of the differences in the expression levels of the PDK2 and PDK4 genes in the liver tissue transcriptomics between IL-33 gene overexpressing mice and wild-type mice using qPCR experiments. (**D**) Analysis of PDK4-interacting proteins using the STRING database. The interactions among IL-33 and other hub genes by STRING. (**E**) Enrichment pathway analysis of PDK4-interacting proteins. (5.7e−06 means 5.7 × 10^−6^) (**F**–**K**) Differences in the expression levels of genes related to glucose metabolism patterns, inflammation, and oxidative stress in the liver tissue transcriptomics between IL-33-gene-overexpressing mice and wild-type mice. Circular represents the WT group; square represents the IL-33 group. * *p* < 0.05 and ** *p* < 0.01 compared to the WT.

To further confirm whether pyruvate dehydrogenase kinase 4 (PDK4) inhibits the activity of the pyruvate dehydrogenase complex (PDH) through phosphorylation, regulating the conversion of pyruvate to acetyl—CoA, and thereby affecting the cellular glucose metabolism pattern and participating in the process of IL-33 regulating liver metabolism, first, we analyzed the transcriptional levels of PDK2 (Figure 3A) and PDK4 (Figure 3B) in the liver tissues of wild-type and IL-33-overexpressing mice by combining liver transcriptomic data and qPCR experiments (Figure 3C). The results showed that the levels of both PDK2 and PDK4 decreased when IL-33 was highly expressed, with a more significant downregulation of PDK4. This suggests that PDK4 may be one of the key molecules through which IL-33 affects liver pyruvate metabolism.

We used the String online database (https://cn.string-db.org/, accessed on 14 April 2026) to analyze the interacting proteins of PDK4 (Figure 3D) and its major enriched pathways (Figure 3E). The results showed that the interacting proteins of PDK4 mainly included Pdha1, Pdhx, Dlat, Hspa9, etc. Further gene enrichment analysis of the interacting proteins revealed that these genes were mainly enriched in processes such as mitochondrial acetyl—CoA biosynthesis and glucose metabolism. Liver glucose metabolism is a core link in maintaining whole-body energy homeostasis. Changes in the liver glucose metabolism pattern are not only the core pathological features of various liver diseases (especially MASLD and liver cirrhosis) but also a key link driving systemic metabolic disorders. Therefore, we further analyzed the changes in the transcriptional levels of genes related to glucose metabolism in the liver tissues of mice with highly expressed IL-33. The results showed that when IL-33 was highly expressed, the expression of genes related to glycolysis (HK2 and PDK) decreased in the liver tissues, while the expression of genes related to OXPHOS (NDUFA1 and MT—CO1) increased. At the same time, the inflammation-related gene Chil3 was significantly downregulated, and the level of the antioxidant stress gene CAT was significantly upregulated (Figure 3F–K). The above results suggest that IL-33 participates in the reprogramming of the liver tissue glucose metabolism pattern and the reprogramming process of the liver immune–inflammatory response through PDK4.

**Figure 4:**
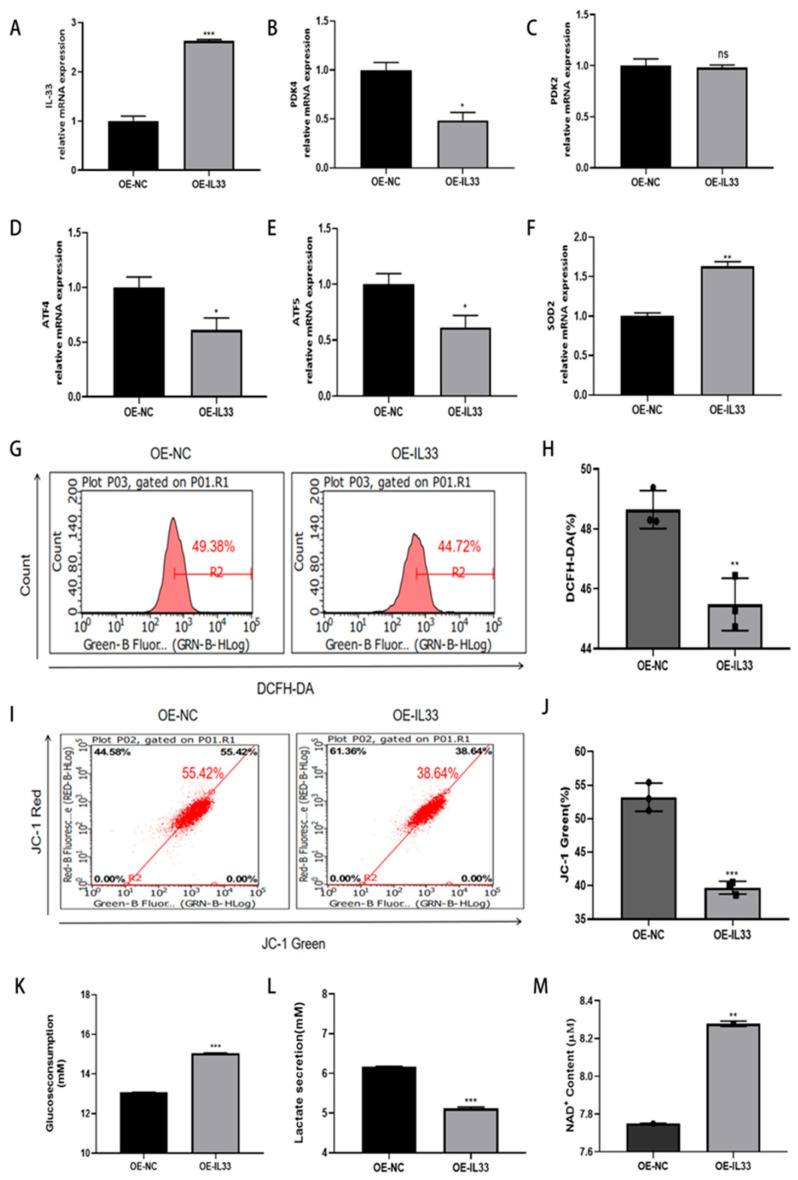
IL-33 promotes mitochondrial oxidative phosphorylation metabolism in mouse hepatocytes. (**A**) After overexpression of IL-33 in mouse hepatocytes, qPCR was used to detect the change in the mRNA expression of IL-33; (**B**) the change in the mRNA expression of PDK4; (**C**) the change in the mRNA expression of PDK2; (**D**) the change in the mRNA expression of ATF4; (**E**) the change in the mRNA expression of ATF5; and (**F**) the change in the mRNA expression of SOD2. (**G**) Flow cytometry was used to detect the change in cellular reactive oxygen species levels after IL-33 overexpression. (**I**) Flow cytometry was used to detect the change in mitochondrial membrane potential levels after IL-33 overexpression. (**H**,**J**) The columnar statistical graphs of (**G**,**I**), respectively. (**K**) The change in glucose uptake in mouse hepatocytes after overexpression of IL-33. (**L**) The change in lactate secretion. (**M**) The change in NAD^+^ content. * *p* < 0.05, ** *p* < 0.01, *** *p* < 0.001, and ns (not significant) compared to the negative control.

Subsequently, we overexpressed the IL-33 gene in mouse liver cells AML12 in vitro and found that after IL-33 overexpression, the transcriptional level of PDK4 was significantly downregulated, while that of PDK2 showed no significant change (Figure 4A–C). The transcriptional levels of stress response-related genes ATF4 and ATF5 were significantly downregulated, while that of SOD2 was significantly upregulated (Figure 4D–F). The effects of IL-33 overexpression on the oxidative stress capacity of hepatocytes were evaluated by detecting the levels of cellular reactive oxygen species (ROS) and mitochondrial membrane potential using flow cytometry (Figure 4G,I). The results showed that after IL-33 overexpression, the level of cellular ROS decreased, the mitochondrial membrane potential increased, and the cellular redox capacity was enhanced. Furthermore, the glucose metabolic pattern of cells after IL-33 overexpression was evaluated by measuring the cellular glucose uptake (Figure 4K), lactate production (Figure 4L), and NAD^+^ content (Figure 4M). The results showed that after IL-33 overexpression, the cellular glucose uptake increased, while the lactate production and NAD^+^ content decreased, suggesting that IL-33 overexpression promoted the oxidative phosphorylation level in mouse hepatocytes. Therefore, this study demonstrated both in vivo and in vitro that IL-33 can regulate liver glucose metabolism through PDK4 and affect liver stress and inflammatory immune responses.

**Figure 5:**
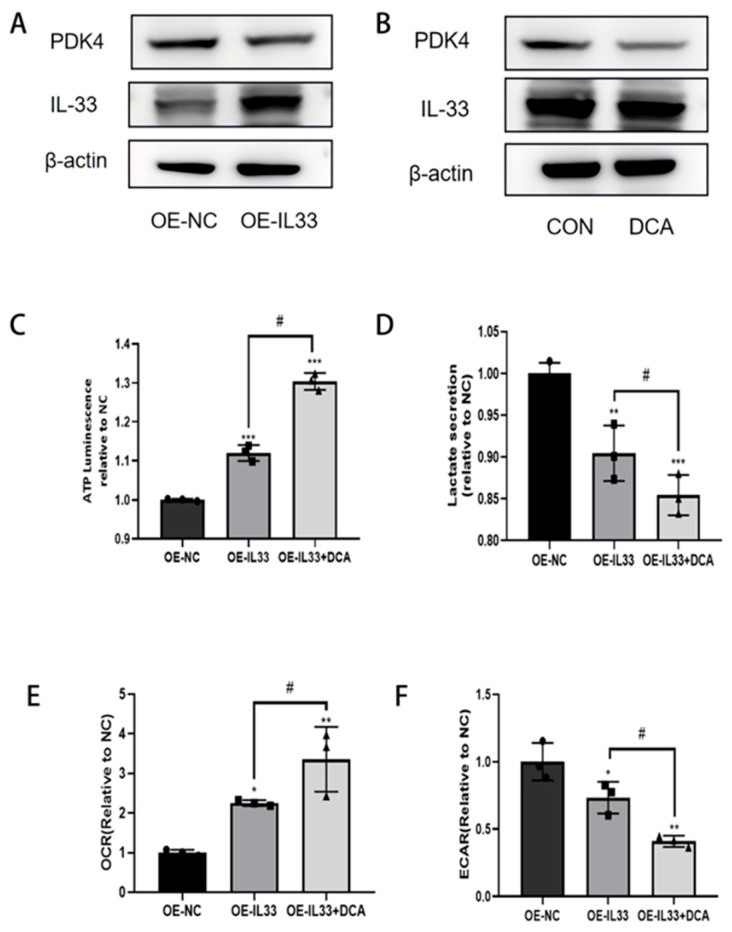
IL-33 promotes mitochondrial oxidative phosphorylation metabolism in mouse hepatocytes. (**A**) After overexpression of IL-33 in mouse hepatocytes, Western blot was used to detect the change in the protein expression of IL-33 and PDK4. (**B**) After the inhibition of PDK4 with DCA, the protein expressions of IL-33 and PDK4 in AML12 cells were assessed using Western blot analysis. After overexpression of IL-33 in AML12 cells combined with DCA to inhibit PDK4, (**C**) ATP production, (**D**) lactic acid levels, (**E**) oxygen consumption rate (OCR), and (**F**) extracellular acidification rate (ECAR) were assessed. * *p* < 0.05, ** *p* < 0.01, and *** *p* < 0.001 compared to the negative control; ^#^ *p* < 0.05 compared to OE-IL33.

To elucidate the relationship between PDK4 and IL-33, we assessed the changes in protein expression levels of IL-33 and PDK4 by overexpressing IL-33 in AML12 cells and inhibiting PDK4 with sodium dichloroacetate (DCA). The results indicated that PDK4 expression decreased following IL-33 overexpression (Figure 5A), whereas no significant alteration in IL-33 expression was observed upon PDK4 inhibition (Figure 5B). Subsequently, we measured variations in cellular ATP production (Figure 5C), lactic acid levels (Figure 5D), oxygen consumption rate (OCR) (Figure 5E), and extracellular acidification rate (ECAR) (Figure 5F). The findings revealed that PDK4 inhibition enhanced the role of IL-33 in regulating glucose metabolism in hepatocytes, as evidenced by increased cellular ATP production and OCR, alongside decreased lactic acid secretion and ECAR. These results further substantiate the influence of the IL-33-PDK4 axis on liver metabolism.

### 3.3. IL-33 Antagonizes Acute Liver Injury in Mice by Promoting Oxidative Phosphorylation

**Figure 6:**
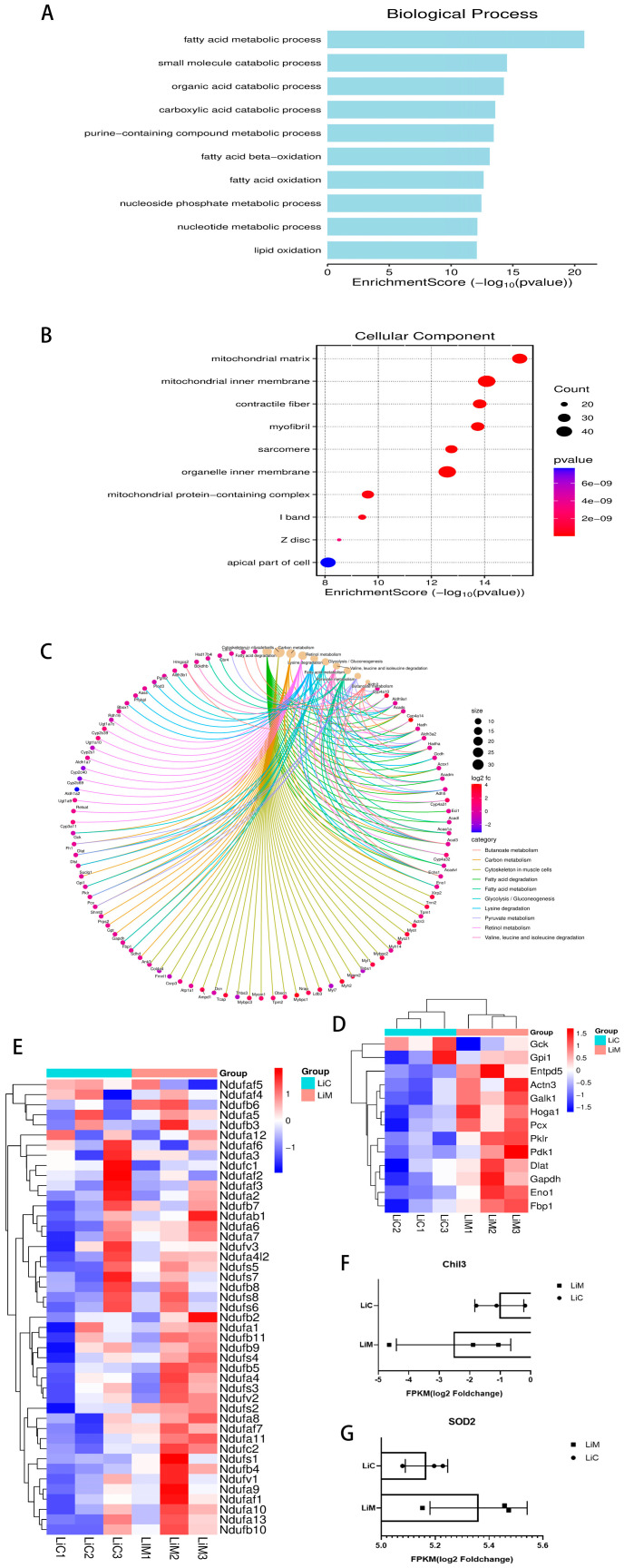
Effects of metformin on glucose metabolism in mouse liver tissue. (**A**) Analysis of the biological processes of differentially expressed genes in liver tissue after metformin treatment in the GSE90755 dataset using the NCBI database [27]. (**B**) Bubble plot of cell components related to differentially expressed genes. The size of the bubble represents the number of enriched genes, and the color of the bubble represents the *p* value (6e−09 means 6.0 × 10^−9^). (**C**) Molecular functions. (**D**) Heat map of the expression of genes related to pyruvate metabolism. (**E**) Heat map of the expression of genes related to mitochondrial complex I. (**F**) Changes in the expression of the inflammatory gene Chil3 after metformin treatment in transcriptomics. (**G**) Changes in the expression of the antioxidant gene SOD2 after metformin treatment in transcriptomics. Circular represents the WT group; square represents the IL-33 group LiC represents liver tissue in the blank control group, and LiM represents liver tissue in the metformin-treated group.

To investigate links between liver disease and glucose metabolism, we analyzed an online NCBI dataset on metformin, a drug that targets hepatic glucose metabolism. Metformin altered liver processes including fatty acid and small-molecule metabolism and produced differential gene expression enriched in mitochondrial compartments such as the matrix and inner membrane. It modulated molecular functions related to fatty acid, glucose, and pyruvate metabolism. Focused analysis of the pyruvate metabolism pathway showed that regulators such as Dlat and Pklr, which occupy central positions in that pathway, dynamically drove marked upregulation of key glucose-metabolism enzymes, affected expression of mitochondrial complex I genes, reduced the inflammatory gene Chil3, and increased the antioxidant gene SOD2 (Figure 6). These results indicate that metformin’s hepatoprotective effects are closely tied to pyruvate-associated glucose metabolism.

**Figure 7:**
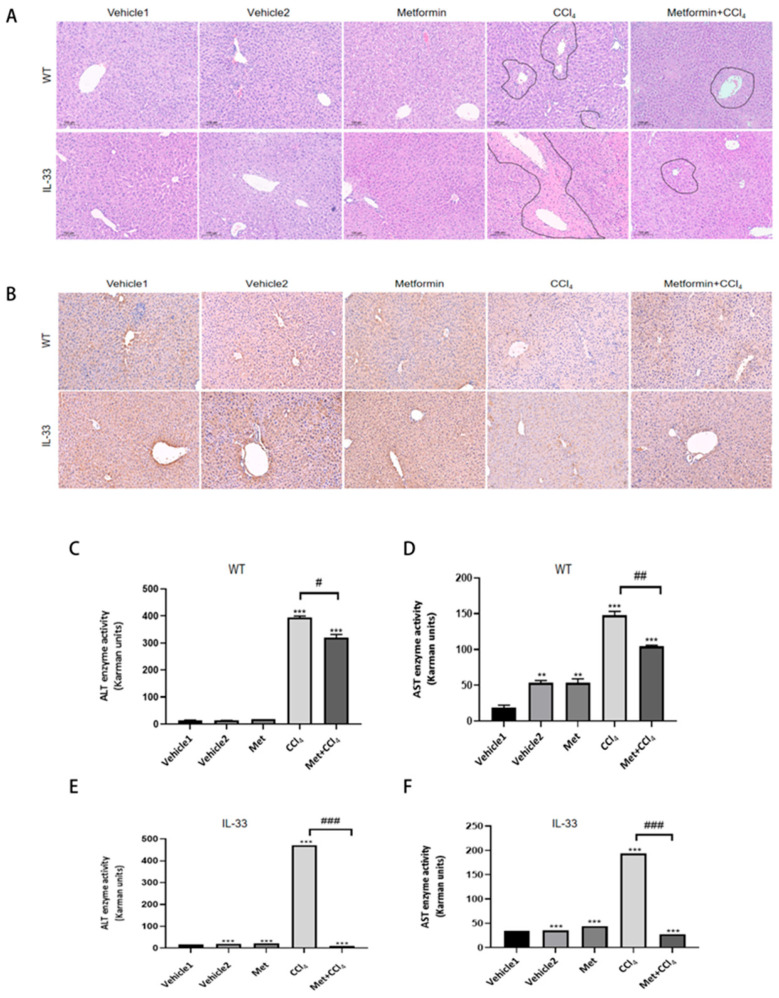
IL-33 is associated with the protective effect of metformin on acute liver injury. (**A**) HE staining images of liver tissues in each experimental group under wild-type and IL-33-gene-overexpression conditions. (**B**) Immunohistochemical staining images of IL-33 expression in each experimental group. (**C**) Expression level of ALT in the serum of wild-type mice. (**D**) Expression level of AST in the serum of wild-type mice. (**E**) Expression level of ALT in the serum of IL-33-gene-overexpression mice. (**F**) Expression level of AST in the serum of IL-33-gene-overexpression mice. Scale bars are 100 μM. Dashed line represents injury border. WT: wild-type C57BL/6J mice. ** *p* < 0.01, and *** *p* < 0.001 compared to the Vehicle 1 group; ^#^ *p* < 0.05, ^##^ *p* < 0.01, and ^###^ *p* < 0.001 compared to the CCl_4_ model group. Vehicle 1: saline, Vehicle 2: olive oil (CCl_4_ solvent), Met: metformin), and CCl_4_: carbon tetrachloride. *n* = 3 per group per experiment.

Using IL-33 gene overexpression mice and a mouse acute liver injury model established by CCl_4_ [28,29], we first determined whether IL-33 can affect mouse liver injury. The results showed that after CCl_4_ treatment, there were peripheral necrotic foci in the large blood vessels of the mouse liver tissue (Figure 7A), and the levels of ALT and AST in the serum increased significantly. After metformin treatment, the peripheral necrotic foci in the mouse liver tissue decreased significantly, and the levels of ALT and AST in the serum were significantly lower than those in the CCl_4_ model group. Among them, the changes in the levels of the above transaminases were more significant in the IL-33-overexpression mice (Figure 7C–F). Further immunohistochemical staining results showed that the expression level of IL-33 in the CCl_4_ model group decreased significantly, while that in the metformin group increased significantly (Figure 7B). The above results indicate that IL-33 is related to the protective effect of metformin on acute liver injury.

**Figure 8:**
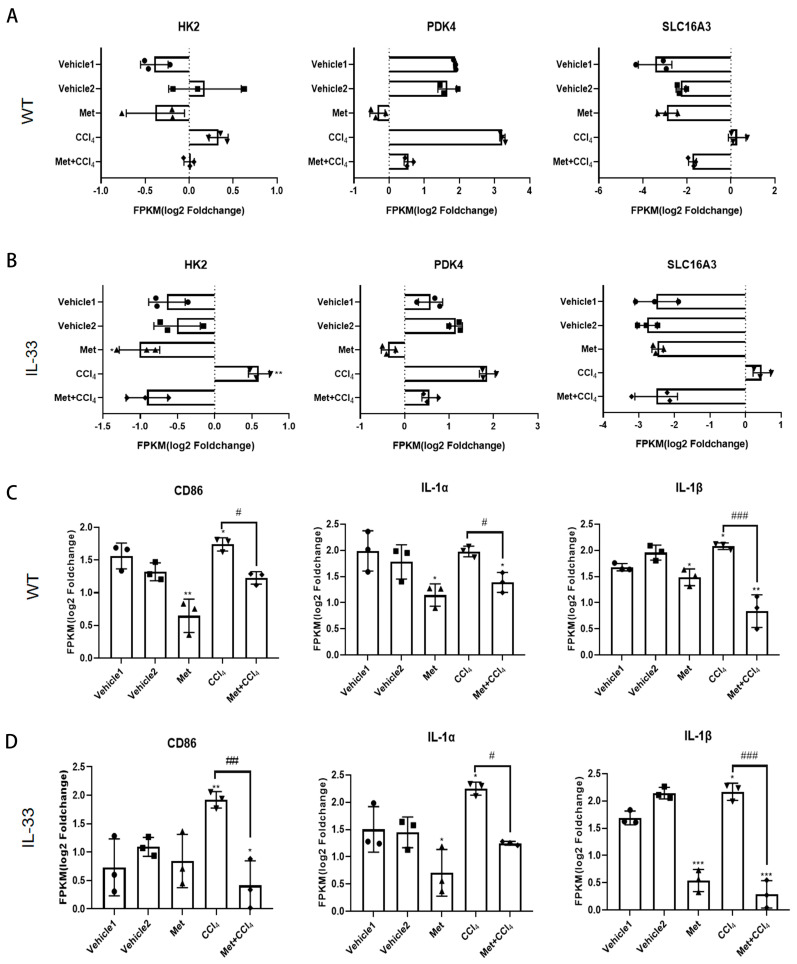
IL-33 antagonizes acute liver injury in mice by promoting oxidative phosphorylation. (**A**) Changes in the expression levels of genes related to glucose metabolism in the liver tissue transcriptomics of mice in each experimental group of wild-type mice. (**B**) Changes in the expression levels of genes related to glucose metabolism in the liver tissue transcriptomics of mice in each experimental group with IL-33 gene overexpression. (**C**) Changes in the expression levels of genes related to inflammation in the liver tissue transcriptomics of mice in each experimental group of wild-type mice. (**D**) Changes in the expression levels of genes related to inflammation in the liver tissue transcriptomics of mice in each experimental group with IL-33 overexpression. WT: wild-type C57BL/6J mice. * *p* < 0.05, ** *p* < 0.01, and *** *p* < 0.001 compared to the Vehicle 1 group; ^#^ *p* < 0.05, ^##^ *p* < 0.01, and ^###^ *p* < 0.001 compared to the CCl_4_ model group. Vehicle 1: saline, Vehicle 2: olive oil (CCl_4_ solvent), Met: metformin, and CCl_4_:carbon tetrachloride. *n* = 3 per group per experiment.

To further verify whether metformin and IL-33 can antagonize CCl_4_-induced acute liver injury by affecting the glucose metabolism pattern, we analyzed the genes related to glucose metabolism in the liver tissue transcriptomic data of wild-type and IL-33-overexpressing mice. We found that after CCl_4_ treatment, the expression of the glycolysis-related gene HK2, pyruvate metabolism-related gene PDK4, and lactate metabolism-.related gene SLC16A3 was significantly increased, while the expression of these genes was significantly downregulated after combined treatment with metformin (Figure 8A,B). The expression of inflammation-related genes CD86, IL-1α, and IL-1β was significantly downregulated in the metformin combined treatment group compared with the CCl_4_ model group (Figure 8C,D). The above results indicate that metformin can promote the expression of IL-33, reduce the glycolysis level in liver tissue, promote the transformation of cellular glucose metabolism to oxidative phosphorylation, reduce the liver inflammatory response, and antagonize CCl_4_-induced acute liver injury.

### 3.4. IL-33 Antagonizes Acute Liver Injury by Promoting M2 Macrophage Polarization

**Figure 9:**
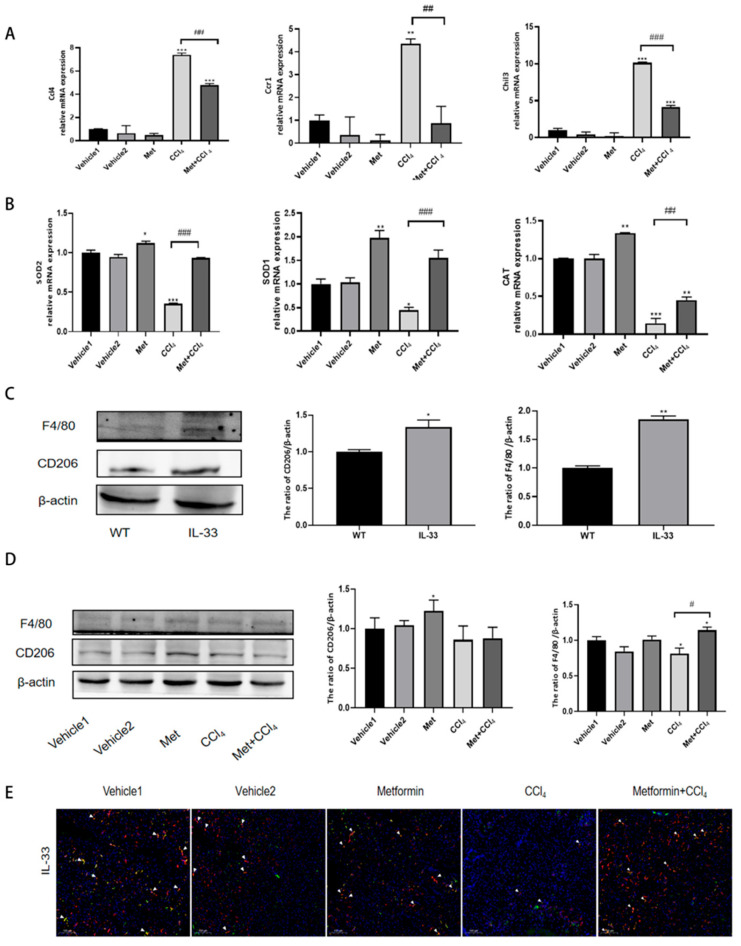
IL-33 antagonizes acute liver injury by promoting M2 macrophage polarization. (**A**) Changes in the expression levels of inflammation-related genes in the liver tissue transcriptomics of mice in each experimental group with IL-33 gene overexpression. (**B**) Changes in the expression levels of oxidative stress-related genes in the liver tissue transcriptomics of mice in each experimental group with IL-33 gene overexpression. (**C**) Western blot detection of changes in the expression of macrophage marker protein F4/80 and M2 macrophage marker protein CD206 in WT and IL-33^+^ mice. (**D**) Western blot detection of changes in the expression of macrophage marker protein F4/80 and M2 macrophage marker protein CD206 in IL-33^+^ mice. (**E**) The tissue staining data in IL-33^+^ mice. Nuclear (blue); F4/80 (red); CD206 (green) and the white arrow indicates the merge area. Scale bars shown are 100 μm. WT: wild-type C57BL/6J mice, IL-33^+^: C57BL/6J mice overexpressing IL-33. * *p* < 0.05 and ** *p* < 0.01 compared with Vehicle I (control group); * *p* < 0.05, ** *p* < 0.01, and *** *p* < 0.001 compared to the Vehicle 1 group; ^#^ *p* < 0.05, ^##^ *p* < 0.01, and ^###^ *p* < 0.001 compared with the CCl_4_ model group. Vehicle 1: saline group, Vehicle 2: olive oil (CCl_4_ solvent), Met (metformin), and CCl_4_ (carbon tetrachloride). *n* = 3 per group per experiment.

To further clarify the correlation between the protective mechanism of the glucose metabolism pattern affected by IL-33 in the liver and the immune inflammatory response, qPCR experiments were first performed to further verify the expression changes of inflammation-related genes Ccl4, Ccr1, and Chil3 (Figure 9A), as well as oxidative stress-related genes SOD1, SOD2, and CAT in the liver tissues of IL-33 gene overexpression mice (Figure 9B). The results showed that the combined treatment with metformin reduced the expression of inflammatory genes and promoted the expression levels of cellular antioxidant stress genes.

There is a close, bidirectional regulatory relationship between liver macrophage polarization and liver injury [30,31]. Central to this relationship is the decisive influence of dynamic shifts between macrophage phenotypes—M1 (pro-inflammatory) and M2 (anti-inflammatory/repair)—on hepatic inflammation, injury progression, and repair. Liver glucose metabolism and macrophage polarization interact through metabolic signals, inflammatory mediators, and key regulators (such as FoxO1, SIRT6, and PGC-1α), together maintaining metabolic homeostasis and shaping disease trajectories. To test whether the hepatoprotective effect of IL-33 on glucose metabolism involves macrophage polarization, we used a Western blot to measure F4/80 and CD206 protein levels in mouse livers after IL-33 overexpression (Figure 9C,D). IL-33 increased CD206 expression, a marker of M2 macrophages, and immunofluorescence staining corroborated the Western blot findings. In the CCl_4_-induced acute liver injury model, combined treatment with metformin also enhanced CD206 expression (Figure 9E). These results indicate that IL-33 promotes M2 macrophage polarization by elevating hepatic oxidative phosphorylation, thereby counteracting acute liver injury in mice.

## 4. Discussion

As an alarm factor of the immune system, current research has found that the function of IL-33 highly depends on cell sources (such as HSCs and endothelial cells), release forms (nuclear storage/cytoplasmic secretion), receptor distribution (ST2^+^ immune cell subsets), and microenvironmental signals [9,32,33]. For example, in a mouse model of drug-induced liver injury induced by acetaminophen (APAP), the IL-33/ST2 pathway plays a liver-protective role by enhancing the interaction between Beclin-1 and STING, promoting the acetylation and autophagic degradation of STING, and inhibiting the type I interferon response mediated by cGAS/STING [34]. Hilal Ahmad Khan et al. found that when constructing an immune-induced mouse liver injury model using ConA, rmIL-33 could significantly reduce the expression of liver transaminase AST/ALT and alleviate liver inflammatory responses [16]. However, in the context of chronic inflammation, fibrosis, and specific liver cancers, it may promote pathological progression. For example, the level of IL-33 increases in the liver tissue and serum of patients with chronic liver disease fibrosis, which promotes the polarization of CD4^+^ T cells towards a Th2-like phenotype and recruits them to the liver. It activates hepatic stellate cells (HSCs) through paracrine IL-13, thus promoting the progression of fibrosis. In this study, by constructing IL-33-gene-overexpression mice, we comprehensively analyzed in the liver tissue, the body’s core metabolic organ, that as an alarmin, IL-33 can influence the glucose metabolism pattern through PDK4 and intervene in liver immune and inflammatory responses in addition to the classical pathway. Through constructing IL-33-gene-overexpression mice and conducting in vivo and in vitro experiments in mouse hepatocyte AML12, we found that after IL-33 gene overexpression, the expression of PDK4 in the pyruvate metabolic pathway was significantly downregulated, which in turn changed the glucose metabolism pattern of hepatocytes, promoted liver oxidative phosphorylation, and affected liver stress, inflammation, and immune responses. Furthermore, the body’s immune response was connected with the glucose metabolism pattern through IL-33/PDK4. In the acute liver injury model constructed with CCl_4_, it was further verified that the liver glucose metabolism pattern regulated by IL-33 could promote hepatocyte oxidative phosphorylation and further promote the expression of CD206, a marker protein of M2 macrophages, the key cells in liver inflammatory responses, thus antagonizing acute liver injury in mice. Previous studies indicate that IL-33 plays a crucial protective role in liver ischemia–reperfusion injury (IRI) and regeneration, through a cascade immune regulatory network characterized by promoting M2 polarization by regulating the IL-33/ILC2s/IL-13 axis. Additionally, some research has demonstrated that the polarization of M2-type macrophages is closely associated with oxidative phosphorylation. In this article, we begin by elucidating the impact of IL-33 on the glucose metabolism patterns of liver cells through liver transcriptomics [35]. We then further investigate the protective effect of these glucose metabolism patterns in CCl_4_-induced acute liver injury by incorporating metformin, a drug that modulates liver glucose metabolism. This approach enhances our understanding of the relationship between alterations in glucose metabolism and macrophage polarization in the context of liver injury.

As a core metabolic organ, the liver plays a crucial role in the regulation of glucose homeostasis (e.g., endogenous glucose production, glycogen storage and breakdown). There is a close and bidirectional pathological association between hepatic glucose metabolism disorders and various liver diseases. Liver diseases lead to insulin resistance and glucose metabolism disorders, while hyperglycemia, hormonal imbalance, and abnormal molecular pathways accelerate liver injury. Liver diseases can cause abnormal glucose metabolism. For example, in metabolism-dysfunction-associated fatty liver disease, hepatic insulin resistance often occurs, manifested as a decreased ability of insulin to inhibit hepatic glucose production (HGP), and there is a negative correlation between hepatic fat content and the ability of insulin-stimulated hepatic glucose uptake (HGU) [36]. However, abnormal glucose metabolism can also promote the progression of liver diseases. For example, persistent hyperglycemia and insulin resistance promote hepatic lipid deposition, inflammation, and fibrosis, accelerating the development of MASLD to MASH, liver cirrhosis, and even hepatocellular carcinoma (HCC) [37,38]. In this paper, when acute liver injury was induced in mice using CCl_4_, it was found that the level of glycolysis increased significantly, indicating that the glucose metabolism pattern is closely related to acute liver injury. Acute liver injury represents a critical juncture in the continuum of liver diseases. It serves not only as the acute endpoint manifestation of various etiologies, including drugs, viruses, and metabolic disorders, but also as a catalyst for the progression of chronic liver diseases. The role of IL-33 in liver diseases is a potential area for further investigation, thereby offering new insights for targeted therapeutic approaches in liver disease research.

Pyruvate is a central intermediate of glucose metabolism that links glycolysis, the tricarboxylic acid cycle (TCA), gluconeogenesis, lipid synthesis, and amino acid metabolism in the liver. Pyruvate flux, the expression of its key enzymes, and dysregulated control networks constitute the core of hepatic energy and substrate conversion and directly contribute to pathologies such as fatty liver, liver injury, and metabolic syndrome. Targeting critical nodes in pyruvate metabolism (for example, PKLR, MPC, and PDH) or using pyruvate itself as a therapeutic intervention offers important avenues for mechanistic studies and treatment development for metabolic liver diseases [39]. The pyruvate dehydrogenase kinase (PDK) family phosphorylates and inhibits the pyruvate dehydrogenase complex (PDH), thereby preventing pyruvate entry into the TCA cycle, promoting a shift toward glycolysis, disturbing hepatic energy homeostasis, and exacerbating inflammation and oxidative stress; these actions are implicated in alcoholic liver disease, septic liver injury, liver cancer, and other metabolism-related hepatic disorders [24,40,41,42]. PDK4, which is highly expressed in liver tissue, is upregulated under diverse metabolic stresses (for example, oxidative stress and drug toxicity). Prior studies indicate that PDK4 inhibition in cardiac tissue can confer cardioprotection by improving mitochondrial function and reducing ferroptosis [42]. In alcohol-related liver disease, the use of dichloroacetic acid (DCA), a pyruvate dehydrogenase kinase inhibitor, can prevent ethanol-induced liver inflammatory responses and metabolic disorders in mice, suggesting that inhibiting PDK activity (including PDK4) may be a potential strategy for the treatment of alcohol-related liver disease [40]. In this paper, we further demonstrated in an acute liver injury model that IL-33 can alter the glucose metabolism pattern of liver cells via PDK4, thereby regulating acute liver injury. Therefore, this study leverages the characteristics of metabolic reprogramming of liver cells with overexpressed interleukin-33 to gain a deeper understanding of the mechanism of metformin’s protective effect on liver cells’ metabolic reprogramming, providing a new theoretical basis for the clinical use of metformin in anti-inflammation and liver protection.

## 5. Conclusions

In conclusion, we have demonstrated that IL-33 is a crucial metabolic checkpoint in the liver, capable of counteracting the glycolytic effect induced by CCl_4_. IL-33 has been identified as a key regulator of mitochondrial metabolism, indicating that the role of this cytokine goes beyond its extracellular “alarmin” activity. For instance, when the liver is under stress, IL-33 helps reduce oxidative damage in liver tissue and promotes mitochondrial metabolism. IL-33 promotes oxidative phosphorylation metabolism by reducing the expression of PDK4, induces M2 macrophage polarization, and counteracts acute liver injury in mice. This provides a new target for targeted metabolic therapy of liver diseases.

### Limitations of the Study

The liver tissue is composed of various types of cells. This paper focuses on the relatively abundant macrophages in the liver tissue, and the roles of other cells in the liver tissue in acute liver injury have not been fully discussed. The IL-33-gene-overexpression mice constructed in this paper have overexpression in all organs of the body. As the liver is a key hub for the regulation of body homeostasis, the changes in liver function are a process of coordinated action among all tissues and organs in the body. In the future, we will conduct further analysis on multiple organs.

## Data Availability

The original contributions presented in this study are included in the article. Further inquiries can be directed to the corresponding author(s).
